# Patch-Level Feature Selection for Thoracic Disease Classification by Chest X-ray Images Using Information Bottleneck

**DOI:** 10.3390/bioengineering11040316

**Published:** 2024-03-26

**Authors:** Manh Hung-Nguyen

**Affiliations:** Faculty of Electrical and Electronics Engineering, HCMC University of Technology and Education, Ho Chi Minh City 7000, Vietnam; hungnm@hcmute.edu.vn

**Keywords:** feature selection, CXR image, classification

## Abstract

Chest X-ray (CXR) examination serves as a widely employed clinical test in medical diagnostics. Many studied have tried to apply artificial intelligence (AI) programs to analyze CXR images. Despite numerous positive outcomes, assessing the applicability of AI models for comprehensive diagnostic support remains a formidable challenge. We observed that, even when AI models exhibit high accuracy on one dataset, their performance may deteriorate when tested on another. To address this issue, we propose incorporating a variational information bottleneck (VIB) at the patch level to enhance the generalizability of diagnostic support models. The VIB introduces a probabilistic model aimed at approximating the posterior distribution of latent variables given input data, thereby enhancing the model’s generalization capabilities on unseen data. Unlike the conventional VIB approaches that flatten features and use a re-parameterization trick to sample a new latent feature, our method applies the trick to 2D feature maps. This design allows only important pixels to respond, and the model will select important patches in an image. Moreover, the proposed patch-level VIB seamlessly integrates with various convolutional neural networks, offering a versatile solution to improve performance. Experimental results illustrate enhanced accuracy in standard experiment settings. In addition, the method shows robust improvement when training and testing on different datasets.

## 1. Introduction

Chest X-ray (CXR) examination is a widely used examination in clinical diagnosis. However, training a qualified doctor who can understand X-ray images is expensive. In many developing countries, ensuring every local hospital has a doctor who can read and understand a medical X-ray imaging is difficult. Therefore, using artificial intelligence (AI) models [1,2,3,4] to simulate the knowledge of diagnosis experts and using them in local hospitals is a solution that has been considered. Ensuring the generality of AI models is the most crucial factor in completing this system.

An AI-based diagnosis system is considered a multi-label classification problem [5,6]. Initial research uses transfer learning to train a deep learning model for the classification task. This method involves reusing or adapting a model trained on one task to improve performance on a related task. Instead of starting from scratch, transfer learning leverages knowledge acquired from a source task to enhance learning on the target task. The knowledge from the source task is represented by parameters in the model, which is called a backbone. This approach is particularly beneficial when there are limited labeled data available for the target task or when training a model from scratch is computationally expensive. In chest X-ray (CXR) image analysis, popular backbone architectures used for transfer learning include AlexNet [7], GoogLeNet [8], DenseNet121 [9], and ResNet [10]. AlexNet, introduced in 2012, is a relatively simple network with five convolutional neural network (CNN) layers but a large number of parameters (60 million). GoogLeNet, introduced in 2014, employs inception blocks to capture multi-scale features and has a deeper architecture compared to AlexNet, with fewer parameters (6.7 million). ResNet utilizes residual learning, where each layer learns a residual mapping with respect to the input by incorporating skip connections, resulting in various depths, such as ResNet-18, ResNet-50, ResNet-101, and ResNet-152. DenseNet introduces dense connectivity patterns, where each layer is connected to every other layer within a dense block, facilitating feature reuse and information flow throughout the network. While the backbones work well in most vision-based applications, CXR image-based diagnosis has its own challenges that must be adequately addressed.

**Figure 1:**
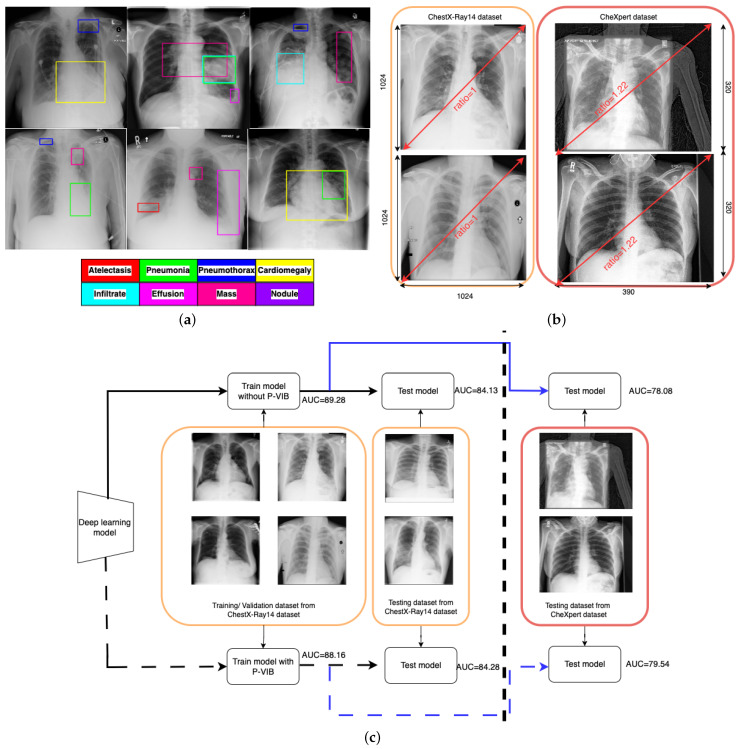
Challenges of CXR image-based thoracic disease diagnosis and the contribution of the proposed method. (**a**) The challenge of multiple lesion areas in a single CXR image. (**b**) Domain gap challenge. (**c**) Contribution of patch-level VIB. Orange boxes contain images from ChestX-Ray14 dataset, and red boxes contain images from ChestExpert dataset; the dashed lines mean experiments with P-VIB, and the solid lines mean experiments without P-VIB; the black lines mean training and validating a model, and the blue lines mean testing a model.

One major challenge of CXR image-based diagnosis is that multiple lesion areas exist in a CXR image, as shown in Figure 1a. In the figure, each box represents a lesion area, and the color corresponds to its respective pathology. This phenomenon occurs because one latent reason may cause several pathologies. The pathology co-occurrence is considered an intrinsic correlation among multiple diseases, and the correlation is modeled by interaction among non-local regions [11]. According to the review of Guo et al. [12], the attention module is a successful solution to learning non-local features. The attention module is a mechanism that allows neural networks to focus on specific parts of the input data (such as words in a sentence or regions in an image) while performing a task. Inspired by the human visual system, the key idea behind the attention module is to assign different weights to different parts of the input data dynamically, allowing the model to attend to the most relevant information selectively. In thoracic disease classification [13,14,15], attention modules help lesion areas in a chest X-ray image interact together and extract a better feature.

In addition to the attention mechanisms, advantage techniques [15,16] were used to enhance accuracy. Typically, an advanced classifier comprises three main components: the backbone, neck, and head. The backbone is responsible for extracting features from an input image, while the neck enhances these features, and the head makes the final classification prediction. In ConsultNet [15], the backbone is DenseNet121, while the neck consists of a two-branch network comprising a feature selector and a feature integrator, and the head comprises a fully connected layer. Specifically, the feature integrator is an attention-based layer designed to capture non-local information, while the feature selector is a variational selective information bottleneck (VSIB) aimed at selecting crucial disease-specific features based on their importance. A vanilla variational information bottleneck (VIB) introduces a probabilistic model aimed at approximating the posterior distribution of latent variables given input data, thereby enhancing the model’s generalization capabilities on unseen data. The VSIB module in ConsultNet [15] builds upon the conventional VIB but introduces a novel selection mechanism. This mechanism generates an importance matrix to specify the significance of each element of the input feature, eliminating the need to approximate the posterior distribution for these critical features. Recently, MLRFNet [16] has greatly improved in CXR image-based diagnosis. This method utilizes Res2Net [17] as the backbone, the ECA module [18] as the neck, and the CSRA module [19] as the head. The ECA module, functioning as an attention mechanism, focuses on capturing non-local features. Meanwhile, the CSRA module serves as the multi-label classification head. Additionally, MLRFNet employs a multi-scale approach to resolve classification challenges, thereby enhancing accuracy. While many promising results have been reported, deploying these models in the operation phase is still an open question. X-ray machines at different medical facilities will vary in calibration and operation. The domain gap can degrade the accuracy of the AI model.

Figure 1b illustrates the domain gap between the ChestX-Ray14 [5] and ChestExpert [6] datasets. In the ChestX-Ray14 dataset, all images have a standardized resolution, with dimensions of 1024 rows and 1024 columns, resulting in a width-to-height ratio of 1.0. Conversely, most images in the ChestExpert dataset have dimensions of 320 rows and 390 columns, leading to a width-to-height ratio of 1.22. During regular training processes, input images are typically resized to specific ratios, such as [512,512] or [224,224]. This resizing approach may not significantly impact the ChestX-Ray14 data due to the consistent width-to-height ratio across both training and original images. However, it could introduce distortions into the ChestExpert dataset, where the original width-to-height ratio has been altered. Additionally, the position of lung regions differs between the two datasets. In the ChestX-Ray14 dataset, the lung region is consistently aligned at the center of the image. Conversely, in the ChestExpert dataset, the lung region is not consistently centered, resulting in some redundancies on the right side of the images. This analysis suggests that the ChestX-Ray14 dataset is better prepared and cleaner compared to the ChestExpert dataset.

In real-world applications, deep learning models are trained and validated using meticulously curated datasets. However, during operational deployment, testing images may differ substantially from those used in training. To simulate such scenarios, we utilize the ChestX-Ray14 dataset for training and validation while employing the ChestExpert dataset for testing purposes. As depicted by the solid line in Figure 1c, the area under the curve (AUC) is 89.28% and 84.13% for the training and validation datasets, respectively. However, due to the domain gap illustrated in Figure 1b, there is a significant reduction in AUC for the testing dataset. Following the blue solid line, the testing AUC is only 78.08%.

To improve accuracy in the operation phase, this paper proposes a patch-level feature selection technique. This method’s core idea is to use a variational information bottleneck (VIB) [20] to select important features at a patch level. Given an input image with dimensions [3,W,H], the backbone extracts features at dimensions $\left[d,W_{c},H_{c}\right]$, where $W_{c}$ and $H_{c}$ are smaller than *W* and *H*, respectively. Hence, each position on the feature map captures information within a small w×w window. In conventional VIB, features are flattened before applying the re-parameterization trick. In this scenario, the feature selection is applied globally on the image. In our method, feature selection is involved at every position within a feature map in a w×w local region (or a patch-level). In addition, unlike the conventional VIB approach [20] or ConsultNet [15], where VIB-based features serve as the input for a classification head, our method can extract VIB-based features at any position in a CNN network, not just at the classification head. Instead of flattening all features and using the VIB method [20] to sample a new latent feature, we apply a convolution block to learn sampling parameters for every position in a feature map. This design allows only important pixels to respond on a feature map, and the model will select important patches in an image. In addition, the innovative design enables seamless integration of the P-VIB module into various CNN-based networks at any position. Consequently, we incorporate it into MLRFNet [16] to enhance accuracy. With the help of the proposed module, our method can work better in various scenarios. In detail, implementing the P-VIB module leads to an improvement in the testing AUC to 79.54%, while the evaluation AUC remains stable at 84.28%. This outcome is depicted by the dashed line in Figure 1c.

In summary, the main contributions of this article are as follows:

- We made a connection between VIB and MLRFNet and proposed a patch-level feature selection to enhance disease diagnosis models based on MLRFNet [16] architecture.
- In the standard scenarios (training and testing datasets are from the same dataset) as defined by Wang et al. [5], the proposed method showed an improvement. If the training dataset and testing dataset have some domain gap, the proposed method avoids overfitting on the training dataset and improves the performance of the testing dataset.
- Unlike conventional VIB [15,20], which is usually used for the input of a classification head, the proposed P-VIB could be used at any position in a network and can be successfully integrated with many different lung disease classification methods.

## 2. Related Works

### 2.1. Disease Classification as a Multi-Label Setting

Initially, works focused on using transfer learning [21] to train a multi-label classifier on the ChestX-Ray14 [5] dataset. Here, deep neuron networks such as AlexNet [7], ResNet [10], and DenseNet [9] were trained by the ImageNet1K dataset [22], which contain 1.2 million images across 1000 categories. Later, these networks were fine-tuned on the ChestX-Ray14 [5] dataset, which only has 112,120 images in 14 categories. In regular fine-tuning, only one category is assigned for an input. Hence, the softmax function is used in the output to ensure that the sum of the prediction vector is one. However, CXR image diagnosis is a multi-label classification in which one input may be assigned to several categories. Therefore, sigmoid functions are used at every node in the output. This setting allows multi-outputs to be set to one. Also, binary cross-entropy loss is used in every node to predict a multiple positive output.

After directly applying transfer learning, researchers try to customize models for better performance. Rajpurkar et al. [23] fine-tuned the modified DenseNet121 [9], creating CheXNet, which outperformed professional radiologists in detecting pneumonia. Chen et al. [24] introduced DualCheXNet, a double asymmetric feature learning network based on ResNet and DenseNet, incorporating feature-level fusion and decision-level fusion in its structure for multi-label classification of thoracic diseases.

Besides ChestX-Ray14 [5], the CheXpert [6] dataset is also well-known. The dataset includes 224,316 images whose resolution is slightly different from ChestX-Ray14 [5]. In addition, this dataset provides uncertainty labels for which the truth label is not confident. Hence, it provides scenarios in which uncertainty can be replaced by positive or negative. In contrast to the large-scale ImageNet1K dataset, where each image is assigned to a single category, both the ChestX-Ray14 and CheXpert datasets contain numerous chest X-ray (CXR) images that exhibit no pathology. It creates an imbalance issue when the number of negative samples is higher than the number of positive samples. Focal loss [25], bias focal loss [16], and weighted cross-entropy loss (W-CE loss) [26] are well-known losses used to address the imbalance in these datasets.

These approaches primarily rely on mainstream deep-learning networks to extract pathological features from CXR images, making them susceptible to image noise and irrelevant regions. However, multiple lesion areas are available in a CXR image, which means the corresponding patient suffers from various diseases in a period. Subsequently, several works have tried to discover the intrinsic correlations among thoracic diseases to improve prediction accuracy, as discussed in Section 2.2.

### 2.2. Co-Occurrence Pathologies Challenge

Both ChestX-Ray14 and ChestExpert datasets report that multiple pathologies may exist in a single CXR image. This means that one latent reason can cause multiple diseases. Some works address the challenge by modeling the correlation among the diseases and using it during a training process, while other works try to learn features that will model the intrinsic correlations of multiple diseases in the latent domain. Section 2.2.1 introduces correlation modeling methods in label space, and Section 2.2.2 introduces methods that model intrinsic correlations in feature space.

#### 2.2.1. Correlation Modeling among Thoracic Diseases

In the realm of multi-label thoracic disease classification, improving the model’s recognition capability involves modeling and analyzing dependencies among thoracic diseases. Graph neural networks (GNNs) gained popularity for their robust ability to model relationships between node data. Hence, it is used to encode the relationship among diseases. Chen et al. [27] introduced a label co-occurrence learning framework based on GNN and CNN models, delving into correlations among pathological features. Lee et al. [28] proposed a hybrid deep learning model (CheXGAT) that integrates CNN and graph convolution neural networks (GNNs), using self-attention to aggregate domain features from graphical structures and enhance potential correlations among thoracic diseases. Jung et al. [29] introduced the FGR-PCAM framework, based on GNNs and CNNs, leveraging graph structure to learn relationships among lesion-specific features in localized regions. To endow models with prior knowledge comparable to professional radiologists in diagnosing thoracic diseases, Chen et al. [26] introduced the Semantic Similarity Graph Embedding (SSGE) module to investigate semantic similarities between images and optimize the feature extraction process. However, these approaches still lack semantic information integration at various extraction steps.

#### 2.2.2. Intrinsic Correlation in Feature Space

While GNN can model the correlation among diseases, training a GNN network is not as easy as training a CNN-based network. An alternative method that addresses the correlation among diseases is learning features that can discover the relationship among multiple lesion areas.

Attention mechanisms have been developed to capture information from non-local dependencies. They have quickly gained success in both NLP and computer vision. It is very natural to recognize that this mechanism is very suitable for modeling the correlation among multiple lesions in a single CXR image. Wang et al. [13] introduced a triple attention network ($A^{3}$Net) that employs a pre-trained DenseNet121 as the backbone network for feature extraction. $A^{3}$Net integrates three attention modules into a unified framework, facilitating channel-level, element-level, and scale-level attention learning. ConsultNet [15] proposed a feature integrator that serves as an attention module. In addition, ConsultNet [15] utilizes a novel variational selective information bottleneck (VSIB) to concentrate on disease-correlated regions. Chen et al. [14] proposed the attention-guided network LLAGnet, which prioritizes discriminative features from lesion locations by combining region-level attention (RLA) and channel-level attention (CLA). Zhu et al. [30] introduced the pixel classification and attention network (PCAN) for simultaneous disease classification and weakly supervised localization, offering interpretability for disease classification. Chen et al. [31] proposed the PCSANet, a network for thoracic disease classification based on pyramidal convolution and a shuffle attention module. Despite the use of attention mechanisms to guide the model’s focus on key features, these methods rely solely on the output of the final feature map from a CNN during classification and do not incorporate semantic information at different levels. This limitation hinders the improvement of classification accuracy. Recently, MLRFNet [16] uses the advantage of EAC [18] as an attention module. Also, multi-scale features have been used to significantly increase the accuracy when evaluating the ChestX-Ray14 dataset.

## 3. Proposed Method

### 3.1. System Overview

**Figure 2:**
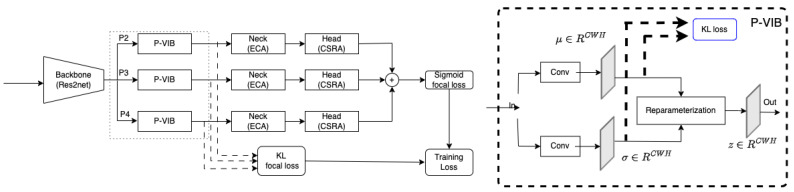
System overview. The solid lines mean connections between modules in a deep network, and the dashed lines mean the connection to loss functions.

This section introduces the system overview of the proposed method. Our method is based on the state-of-the-art (SotA) MLRFNet [16]. The backbone is the Res2Net [17] network; features are extracted at different scales at P2–P3–P4. For each scale, ECA [18] serves as a neck, and CSRA [19] is the multi-label classification head. The classification loss is the bias focal loss in MLRFNet [16]. The left side of Figure 2 summarizes the system overview of the proposed method.

Our modification is the P-VIB module integrated between the backbone and the ECA neck. This module aims to select features at the spatial position level on the extracted feature map. This module is represented on the right side of Figure 2. Here, a KL loss is used to select critical features at a patch level based on information bottleneck [32] theory.

Section 3.2 introduces the concept of feature selection in a multi-label classification task, and Section 3.3 explains how VIB is adopted to select features at the patch level.

### 3.2. Feature Selection in a Multi-Label Classification Task

In Figure 2, the model is trained by using a multi-label classification loss and a feature selection loss. In addition, each loss has three components corresponding to three feature levels.

Denote ${\hat{P}}_{l}$ as the predicted confidence score, which is normalized by a sigmoid layer for each classification head; the classification loss is defined as in Equation (1). The final classification loss for all scales is $Loss^{cls}\left(y,\hat{P}\right)=\sum_{l=1}^{3}Loss^{cls}\left(y,{\hat{P}}_{l}\right)$.

(1) $$Loss^{cls}\left(y,{\hat{P}}_{l}\right)=\frac{1}{C}\sum_{c=1}^{C}-\alpha {\left(1-{\hat{P}}_{l,c}\right)}^{\lambda_{l}}y_{c}\log{\hat{P}}_{l,c}-\left(1-\alpha \right){\hat{P}}_{l,c}^{\lambda_{l}}\left(1-y_{c}\right)\log\left(1-{\hat{P}}_{l,c}\right)$$

Here, *C* is the number of classes, α is a hyperparameter that regulates the bias between negative loss and positive loss, and $\lambda_{l}$ is employed to emphasize challenging samples.

Denote Φ as the parameters of the P-VIB module which are introduced in Section 3.3; $\mu_{l}=\mu_{\Phi ,l}\left(x\right)$ and $\sigma_{l}=\Sigma_{\Phi ,l}\left(x\right)$ are the mean and variance extracted at the *l*th scales given by the P-VIB. The feature selection loss at the *l*th scale is $Loss_{l}^{fea}\left(\mu_{l},\sigma_{l}\right)$, as defined in Equation (2).

(2) $$Loss_{l}^{fea}\left(\mu_{l},\sigma_{l}\right)=KL\left(N\left(z_{l};\mu_{l},\sigma_{l}\right)\left|\right|q\left(z_{l}\right)\right)$$

Here, $N\left(z_{l};\mu_{l},\sigma_{l}\right)$ is the re-parameterization trick as shown in Figure 2, $q(z_{l})$ is a pre-defined distribution representing the approximated latent marginal, and KL(.) is the Kullback–Leibler divergence. Typically, $q(z_{l})$ is a standard normal distribution as $N\left(z_{l};0,I_{k}\right)$. Finally, the feature selection loss for all scales is estimated as Equation (3).

(3) $$Loss^{fea}\left(\mu ,\sigma \right)=\sum_{l=1}^{3}Loss_{l}^{fea}\left(\mu_{l},\sigma_{l}\right)$$

Denote β as a hyperparameter to control the contribution between feature selection loss and multi-label classification; the model is trained end-to-end using the loss function $L(y,\hat{P},\mu ,\sigma )$ in Equation (4).

(4) $$L\left(y,\hat{P},\mu ,\sigma \right)=Loss^{cls}\left(y,\hat{P}\right)+\beta Loss^{fea}\left(\mu ,\sigma \right)$$

### 3.3. Feature Selection at the Patch Level

VIB has been applied in medical image diagnostic in ConsultNet [15]. In the method, the encoder extracts a feature tensor $Z\in R^{NC}$. Here, *N* is the sum of spatial positions over a single channel, and *C* is the number of channels. While promising results have been reported, it raises the issue that flattened features may not be easy to combine with other CNN-based modules. Therefore, in ConsultNet [15], the VIB-based features are fed to a classification head. The limitation of where the VIB module is applied may reduce the flexibility of a deep neural network (DNN).

Recently, many advantage modules have been developed to help increase the accuracy of a CNN network. For instance, successful attention modules such as SE [33] or ECA [18] have been proposed, and they can capture very good non-local features. It raises the question of whether we could customize the vanilla VIB so that it could be used with the advantaged modules.

Motivated by the observation, the P-VIB is proposed in Figure 2. Given a feature $F^{l}\in R^{C^{l}W^{l}H^{l}}$ at the *l*th scale, the P-VIB uses two parallel 3×3 convolution blocks (padding = same) to extract $\mu^{l}\in R^{CW^{l}H^{l}}$ and $\sigma^{l}\in R^{CW^{l}H^{l}}$. Unlike the flatten operator in ConsultNet [15], a convolution operator can retain the shape of the input tensor; hence, the output can be integrated with any CNN-based module. Moreover, the convolution operator can also use neighbor pixels to extract $\mu^{l}$ and $\sigma^{l}$ at every position.

To select features, the term $Loss^{fea}\left(\mu ,\sigma \right)$ in Equation (3) must be estimated via component losses at every *l*th scale as in Equation (2). Given the mean $\mu^{l}$ and variance $\sigma^{l}$ at the *l*th scale, the KL loss at the *l*th scale is estimated by Equation (5). Here, the KL loss $Loss^{fea}\left(\mu ,\sigma \right)$ is estimated at every *j*th position and every *C* channel. This setting may extract sparse features and increase the discriminative level of the learned kernels.

(5) $$Loss_{l}^{fea}\left(\mu_{l},\sigma_{l}\right)=\frac{1}{C_{l}W_{l}H_{l}}\sum_{j=1}^{W_{l}H_{l}}\sum_{k=1}^{C_{l}}\left(\mu_{j,k}^{2}+\sigma_{j,k}^{2}-2log\left(\sigma_{j,k}\right)-1\right)$$

where:

- $C_{l}$ is the dimension of the latent features;
- $W_{l},H_{l}$ are the numbers of rows and columns in the extracted feature map at the *l*th scale;
- *j* is the position’s index;
- *k* is the channel’s index.

The output of the P-VIB module at the *l*th scale is a feature tensor $z^{l}\in R^{CW^{l}H^{l}}$ as a result of a re-parameterization given by Equation (6). Here, the sampling is applied at every spatial position *j* on a feature map with ϵ∼p(ϵ)=N(0,I).

(6) $$z_{j,k}^{l}=\mu_{j,k}^{l}+\epsilon \sigma_{j,k}^{l}$$

## 4. Dataset and Experiment Setting

ChestX-Ray14 [5] is a well-known dataset for thoracic disease classification. It comprises 112,120 frontal-view X-ray images of 30,805 (collected from the years 1992 to 2015) unique patients with 14 common disease labels, text-mined from the radiological reports via NLP techniques. It expands on ChestX-Ray8 by adding six additional thorax diseases: edema, emphysema, fibrosis, pleural thinning, and hernia. If one image has no pathologies, it is considered a No Finding. In addition, each image is assigned one or more of the 14 pathologies, and 880 images have been annotated with 984 labeled bounding boxes for 8 pathologies. The training dataset, testing dataset, and evaluation dataset have been defined in Wang et al. [5], which make it a standard setting used by many research works. Detail of the pathologies and their abbreviations are shown in Table 1.

Motivated by the observation, this work also uses the ChestX-Ray14 [5] dataset to evaluate the proposed P-VIB network. We evaluate performance on all 14 labels. For fairness, the dataset split in the comparative experiments strictly follows the official splitting standards of the dataset published by Wang et al. [5].

To evaluate the generalization of the model, which is trained by ChestX-Ray14 [5], we also use the testing dataset from another dataset. For this paper, CheXpert [6] was selected for the evaluation. The dataset contains 224,316 X-ray scans of 65,240 patients, with 14 observations extracted from the medical reports. Each observation is assigned a positive label in our experiments. Because the 14 pathologies in ChestX-Ray14 [5] are not the same as the 14 pathologies in CheXpert [6] dataset, only 5 observations are selected for examination. These observations are Atelectasis, Cardiomegaly, Consolidation, Edema, and Pleural Effusion, as recommended by Irvin et al. [6].

A thoracic disease diagnosis model is evaluated by the area under the curve (AUC) of the receiver operating characteristic (ROC). This curve is a set of (sensitive and specificity) pairs, and the area under the curve is better than accuracy in terms of evaluating the confidence of a classification task. A smaller AUC represents the performance of a random classifier, and a greater AUC would correspond to a perfect classifier (e.g., with a classification error rate equivalent to zero). Originally, the AUC-ROC curve was only for binary classification problems. However, it can be extended to multi-class classification problems using the One vs. All technique. The average of AUC represents a unique evaluation metric in a multi-label classification setting.

We follow the hyperparameter setting in MRLFNet [16] to train the model. Details of the hyperparameter settings can be found in Table 2. However, our patch size is set as 64 due to the availability of hardware.

In all experiments, early stopping is used to select the best model. This technique applies an evaluation process that estimates the AUC metric on the evaluation dataset for every training epoch. The highest AUC is stored during the training phase. If the AUC in a current epoch is higher than the recent highest AUC, the model at the current epoch is considered the best model, and the current AUC updates the highest AUC. Because the model is trained to fit with the training dataset, the training loss and training AUC will be better at later epochs. Hence, an overfitting may occur at later epochs when the model is trained too long. Applying early stopping allows us to select the best model for the evaluation dataset instead of the last model that may be overfitted to training data.

## 5. Experiment

### 5.1. Hyperparameter Turning

In this section, we address selecting a suitable β hyperparameter. Also, an analysis of the feature selection loss is included to discuss how it can affect the learning process.

**Figure 3:**
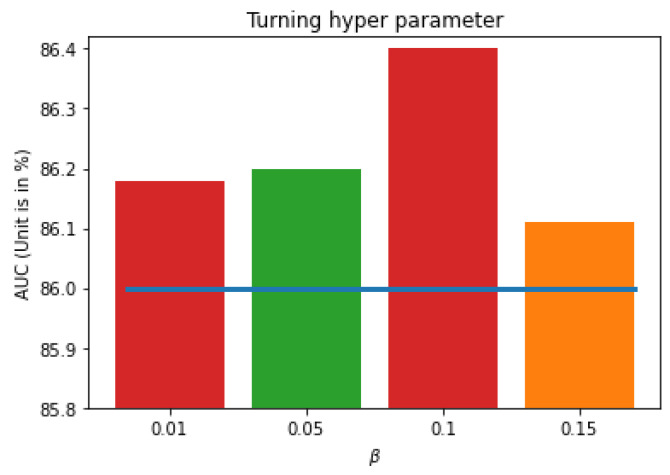
Turning β parameters to get a higher AUC. The blue line is the baseline without P-VIB. The experimental setting follows [5].

In Equation (4), the feature selection loss is accompanied by the classification loss for training. The β parameter controls the contribution of feature selection loss compared to the classification loss. In theory, if we set β too large, the model will select less information and reduce the accuracy. In contrast, if we set the β value too low, the model will not compress the feature, and overfitting may occur. To select a suitable β value, we try several options in the list [0.01,0.05,0.1,0.15]. The micro AUC values given by different β are shown in Figure 3. The result shows that the patch-based KL loss can help to improve the performance. If β=0.1, the micro average AUC of the proposed method is 86.4% compared to the 86.0% reported by the baseline [16]. Moreover, all test cases provide AUCs higher than the baseline. This means the auxiliary loss is very robust to enhance the performance. In addition, because the AUC given by β=0.15 is smaller than the result given by β=0.1, we select β=0.1 in our next experiments.

**Figure 4:**
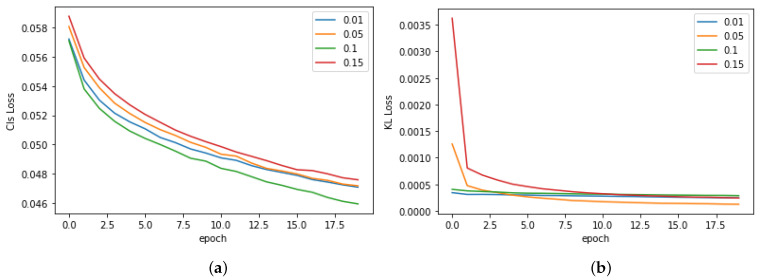
Losses during the training process. (**a**) Classification loss on the training set. (**b**) Feature selection loss on the training set.

The method uses two losses (classification loss in Equation (1) and feature selection loss in Equation (3)) to train the model. Hence, it is valuable to know how each loss contributes to the training process. To address the question, we visualize the classification loss (CLS loss—Equation (1)) and feature selection loss (KL loss—Equation (3)) during a learning process, as in Figure 4. We can see that both losses converge smoothly. However, while classification loss reduces slowly, the feature selection loss drops significantly in the first epoch and converges at similar values. This phenomenon shows that the feature selection loss does not rely too much on an initial state. As shown in Figure 4b, even if the KL loss is very high at the beginning of a training process, it can converge similarly at other settings. Moreover, we may see that feature selection loss and classification loss are quite independent. A smaller KL loss does not mean a smaller CLS loss. For instance, the AUC given by β=0.01 and β=0.05 are similar, and their CLS losses are also similar during a training process. However, their KL losses converge in different manners. These observations point out that, while the feature selection loss robustly improves the performance, it still has many random factors during the training process. Therefore, it is not guaranteed to say β=0.1 is always the best option. Fortunately, a comparison to the baseline in Figure 3 shows that the proposed method can help in all β. This means that, while many uncertainties may remain about how to select the best β, the proposed feature selection loss always helps the training process.

**Figure 5:**
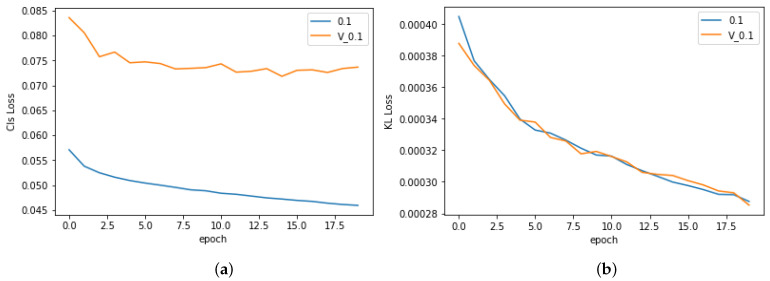
Losses given by β=0.1. The blue color means training loss, and the orange color means validation loss. (**a**) Classification loss on the training set and the validation set. (**b**) Feature selection loss on the training set and the validation set.

In Figure 3, the AUCs are given by the best models for each experiment. As mentioned in Section 4, the best model is selected using the early stopping method. This method is based on the performance during a training process to select the model that works well on the validation set and avoids overfitting on the training set after a long training time. Hence, we visualize the CLS loss and the KL loss on both training and validating datasets, as in Figure 5. Here, the KL loss and CLS loss on the training set and validation set given by β=0.1 are introduced. The result shows that the best classification loss performance is at the 14th epoch. During the training process, the CLS loss on the validation dataset slightly increases from epoch 15 to epoch 20. In contrast, the CLS loss of the training dataset is reduced smoothly. It means overfitting had occurred on the CLS loss. In the case of feature selection loss, its training and validation loss are similar. It means the proposed feature selection loss robustly works and has no negative effect on the training process.

### 5.2. Comparison with SotA for Disease Classification

This section compares our proposed method with SotA methods such as SSGE [26], LLAGNet [14], CheXGCN [27], $A^{3}$Net [13], ConsultNet [15], CheXGAT [28], MXT [34], PCAN [30], PCSANet [31], and F-PCAM [29]. Methodologies of these methods and our proposed method are compared in Table 3. Five factors for comparison are shown as follows:

- Intrinsic correlations: This factor describes how a method addresses the intrinsic correlations among pathologies. Correlation modeling (CM) means a GCN is used to model the correlation. Attention (ATT) means attention modules are used to extract correlation features. In the CM method, the neck is a graph-based fusion module to fuse features. In the ATT method, the neck is an attention module to extract features.
- Backbone: The pre-trained model used in a method.
- Head: The head for multi-label classification. FC means fully connected, CNN means convolution neural network, CSRA [16] means class-specific residual attention, and MB means multi-branch classifier.
- Loss: Training loss. W-CE means weighted cross-entropy loss, BCE means binary cross-entropy loss, FL means focal loss, KL means feature selection loss, and BFL means bias focal loss.
- Scale: The method uses single-scale (S) or multi-scale (M) features for classification.

In this experiment, the accuracy and speed are compared. Macro AUC is used as a well-known metric to evaluate the CXR diagnosis in terms of accuracy; also, floating-point operations per second (FLOPs) and the processing time for one single image are used to measure the speed of a system.

**Figure 6:**
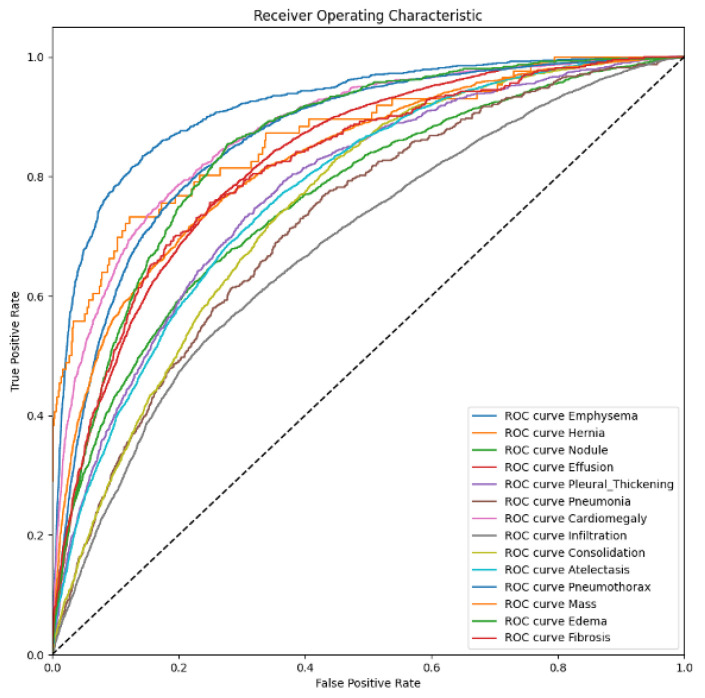
ROCs of our proposed method.

**Figure 7:**
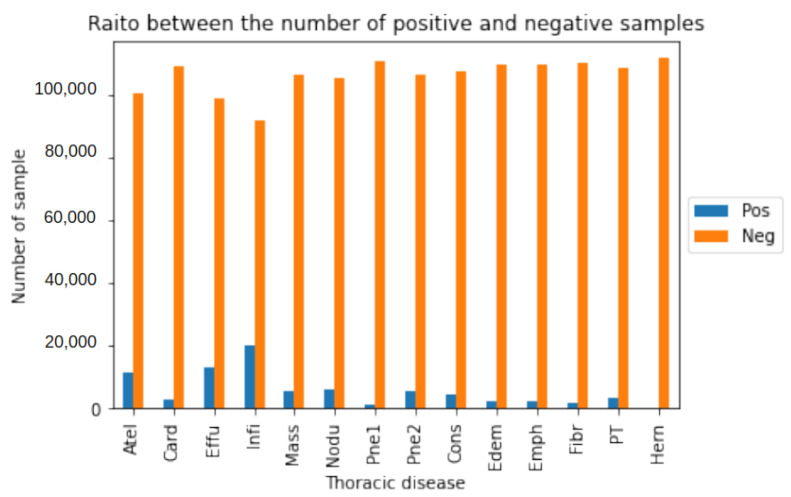
Ratio between the number of positive/negative samples in the ChestX-Ray14 [5] dataset.

Table 4 presents the AUC comparative results obtained by our P-VIB and other SotA baselines; the receiver operating characteristics (ROCs) of the proposed method are shown in Figure 6. Some observations from the table are as follows:

(1) Our approach demonstrates a superior balance compared to other methods. Specifically, our method achieves a higher average AUC than others, even though it excels in performance for only three thoracic diseases (Infiltration, Mass, and Pneumothorax). In contrast, PCSA-NET [31] attains the best AUC for five thoracic diseases (Atelectasis, Cardiomegaly, Effusion, Consolidation, and Edema), but its average AUC is only 0.825. This occurrence arises from SSGE [26] not exhibiting remarkable results for other diseases. Through the incorporation of the proposed feature selection loss, our method effectively mitigates overfitting on certain diseases. The results indicate that our method ranks second-best in 7 out of 14 diseases, leading to an average AUC of 83.7%.
(2) In multi-label classification tasks, the proportion of positive samples plays a pivotal role in assessing the complexity of the task. To gauge the relationship between the ratio of positive to negative samples and the AUCs, we visualize this ratio in Figure 7 and correlate it with the AUC values in Table 4. Analysis of Figure 7 and Table 4 reveals that the pathology with the lowest AUC (Infiltration) exhibits a higher number of positive samples. Despite most methods reporting notably low AUCs for the Infiltration pathology in Table 4, Figure 7 demonstrates that this pathology possesses a greater number of positive samples and fewer negative samples compared to others, suggesting its diverse nature and potential dataset coverage issues. In this context, our proposed method outperforms others due to the re-parameterization mechanism (Equation (6)), which introduces uncertainty into the latent space, akin to a data augmentation technique but in the feature domain, thereby enhancing model robustness. In this scenario, our method surpasses the second-best approach by 2.25% in terms of AUC.
(3) In our approach, the performance of the Hernia pathology stands in stark contrast to that of the Infiltration pathology. While our method achieves an AUC of 0.915 for the Hernia pathology, most other methods achieve higher values. Furthermore, the Hernia pathology exhibits a sparse distribution, with very few positive samples (227 samples) and numerous negative samples (111,893 samples). These statistics indicate that Hernia is a pathology with limited representation in the dataset, suggesting potential overlap between the testing and training sets. In such a scenario, introducing uncertainty into the latent domain may not guarantee improved accuracy.

In addition to evaluating AUC, we compared processing speed between our proposed method and other state-of-the-art (SotA) approaches. Table 5 provides insights into the floating point operations per second (FLOPs) and processing time required for a single image. Our experiments were conducted using an RTX Titan GPU for computation. The results indicate that our method has a higher FLOPs count compared to PCAN and PCSANet, but lower than SSGE, CheXGCN, and LLAGNet. Notably, in our testing environment, the processing speed for a single image is sufficiently fast to support diagnostic applications.

### 5.3. Generalization Evaluation

This section discusses how the proposed method can prevent overfitting on the training dataset. If one model overfits its training data, it may not work well on a testing dataset. Hence, we train the model with data from one dataset and test it with data from another. In addition, if the training dataset size is too small, it also leads to overfitting and can not be generalized enough to work. Therefore, we evaluate our method with various dataset sizes.

In this experiment, we opted to assess the performance of the model trained on the ChestX-Ray14 dataset [5] using a subset of observations from the testing set of CheXpert [6]. Specifically, we focused on five observations: Atelectasis, Cardiomegaly, Consolidation, Edema, and Pleural Effusion.

Let $D_{\mathrm{train}}\left(.\right)$ represent the training dataset and $D_{\mathrm{val}}\left(.\right)$ denote the validation dataset, as specified in Wang et al. [5], tailored specifically for the five selected observations. Additionally, we introduce sub-datasets $D_{\mathrm{train}}^{25}\left(.\right)$, $D_{\mathrm{train}}^{50}\left(.\right)$, $D_{\mathrm{train}}^{75}\left(.\right)$, and $D_{\mathrm{train}}^{100}\left(.\right)$, sampled from 25%, 50%, 75%, and 100% of $D_{\mathrm{train}}\left(.\right)$, respectively. The testing dataset, denoted as $D_{\mathrm{test}}\left(.\right)$, is defined in CheXpert [6]. Throughout all experiments, early stopping is implemented to select the optimal model.

**Figure 8:**
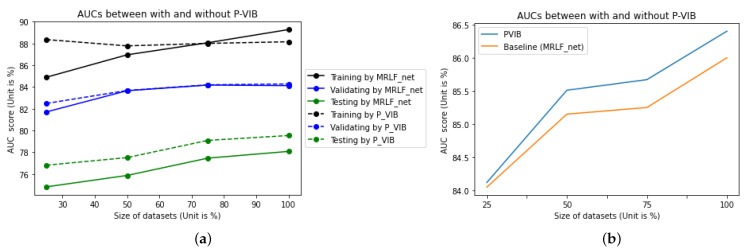
Contribution of the proposed method. The baseline is the SotA MLRFNet [16]. (**a**) Train and validate on the ChestX-Ray14 dataset; test on the CheXpert dataset. Five common diseases are selected. (**b**) Train, validate, and test on the ChestX-Ray14 dataset; all pathologies are selected.

**Figure 9:**
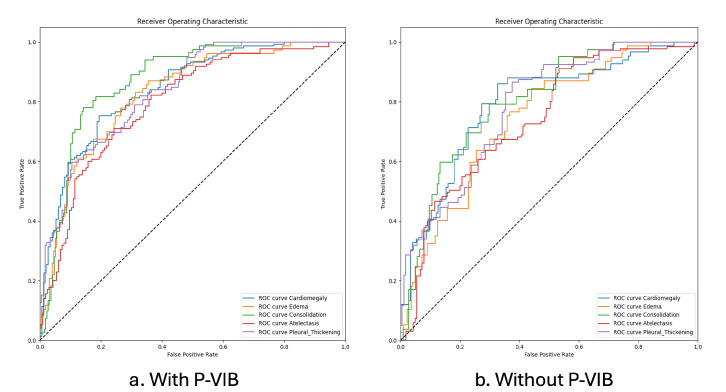
ROC curves for five observations. The model was trained on the ChestX-Ray14 dataset and tested on the CheXpert dataset.

The results are presented in Table 6 and Figure 8a. It is observed that, while P-VIB shows a slight improvement over the baseline on the validation dataset, the significance of the improvement becomes more pronounced on the testing set. This phenomenon indicates a domain gap between ChestX-Ray14 [5] and CheXpert [6]. Without VIB, the best model selected by $D_{\mathrm{val}}\left(.\right)$ exhibits a higher AUC for the training set, but the AUC for $D_{\mathrm{val}}\left(.\right)$ remains the same. This suggests that P-VIB helps prevent overfitting on the training set. Additionally, the results for the $D_{\mathrm{test}}\left(.\right)$ dataset demonstrate that features learned by P-VIB are more suitable for CheXpert [6]. P-VIB contributes to a 2% increase in AUC compared to the baseline when a small dataset ($D_{\mathrm{train}}^{25}\left(.\right)$) is used for training. With a larger dataset ($D_{\mathrm{train}}^{100}\left(.\right)$), the increment is 1.46%. This result proves that P-VIB can help to achieve better generalization and improve the accuracy of smaller datasets. The ROC for each observation with and without P-VIB is shown in Figure 9.

In addition, to better understand the generalization of the proposed P-VIB, we tested all pathologies in the ChestX-Ray14 [5] dataset with different training ratios. Here, the training dataset and testing dataset are established in prior works [5,15,16]. We used sub-training datasets to evaluate how the number of training samples affects the micro AUC. The result in Figure 8b shows that, if more training samples are applied, the increment given by P-VIB is higher. The observation is a little different from the result in Figure 8a where the increment is quite the same with different numbers of training samples. This means that the increment given by P-VIB is dependent on many factors, such as the complexity of the problem and the domain gap issue. However, a bright observation is that P-VIB can help in all scenarios.

### 5.4. Working with Other Networks

This section explores the versatility of applying the P-VIB module across various CNN-based networks. Firstly, Section 5.4.1 investigates how the P-VIB module interacts with different backbone architectures. Secondly, Section 5.4.2 examines the performance of P-VIB when positioned at various locations within a classification network. Lastly, Section 5.4.3 presents an ablation study to evaluate the impact of the P-VIB module across diverse network architectures.

The experiment utilizes two datasets: the first comprises all training images listed in Wang et al. [5], while the second contains 50% of the first case. For consistency, the testing dataset, as defined in Wang et al. [5], remains unchanged across all scenarios. Also, micro AUC is used to evaluate the result.

#### 5.4.1. Working with Different Backbones

Experimental results given by different backbones are listed in Table 7. From these experiments, some observations are as follows:

- P-VIB consistently enhances performance across all scenarios. With a well-optimized backbone like Res2Net, the improvement is modest, with a mere 0.4% increase in micro AUC. However, when the backbone is suboptimal, the enhancement can be more substantial, ranging between 1.3% to 1.6% in AUC.
- Let $\Delta_{VIB}$ denote the improvement attributed to P-VIB, calculated as $AUC_{withP-VIB}-AUC_{w/oP-VIB}$. It is notable that the improvement observed with 100% of the dataset is slightly higher than that with 50% of the dataset. This trend aligns with the observations from Figure 8b, where the enhancement tends to increase with more training samples. However, Figure 8a suggests a slight reduction in improvement as the number of training samples grows. This experiment underscores that the efficacy of P-VIB’s enhancement is not solely contingent on the number of training samples.

#### 5.4.2. P-VIB at Different Positions

**Figure 10:**
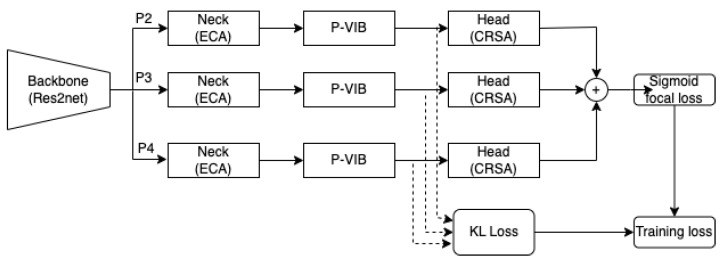
Model structure when the P-VIB module is positioned after the neck.

While the proposed method recommends the utilization of the P-VIB module before the neck, it is important to note that P-VIB can be applied at any position within a deep neural network. In our approach, we refrain from integrating P-VIB into the backbone to maximize the utilization of pre-trained models. Therefore, P-VIB is positioned either before, as illustrated in Figure 2, or after the neck, as depicted in Figure 10. The two settings correspond to the following two learning methods:

- VIB-Neck: Employing feature selection at the level of individual pixels and subsequently leveraging these features to capture non-local features.
- Neck-VIB: Attempting to extract non-local features from all pixels and then selecting new features from these long-range relationships.

**Figure 11:**
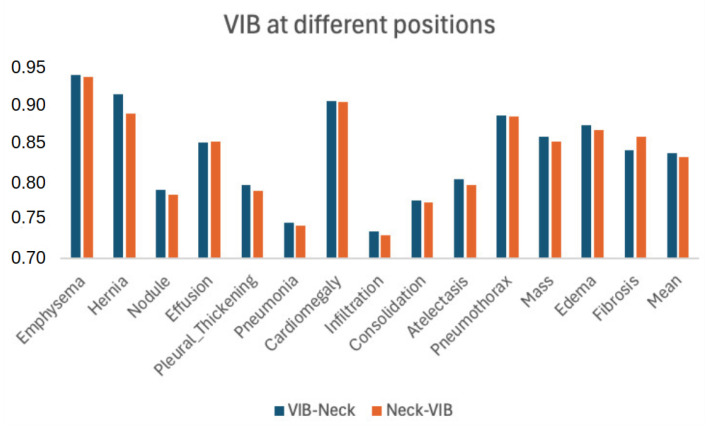
Performance when VIB is used at different positions in a CNN network.

Figure 11 compares results obtained from two settings across all diseases. Both approaches demonstrate successful learning of a classifier, indicating that end-to-end learning is capable of adjusting parameters for both feature selection and non-local fusion regardless of their order of execution. Moreover, the VIB-neck configuration outperforms the neck-VIB arrangement across all diseases except for Fibrosis. This outcome suggests that feature selection is more effective when applied to low-level features rather than high-level features.

#### 5.4.3. P-VIB and Diverse Network Architectures

In this section, the P-VIB module is integrated with diverse network architectures. Initially, the ECA neck is removed to assess the impact of the proposed P-VIB on the network. Subsequently, the scaling process is eliminated, implying that only features at the head P4 are employed for model estimation. Lastly, both the neck and scaling factors are removed.

The results presented in Table 8 indicate that the neck has a minimal effect on accuracy. Without the neck, the AUC decreases slightly compared to the proposed method. When 100% of the training samples are used for training, the AUC is reduced by 0.2% and 0.4% with and without P-VIB. On the contrary, multi-scale features prove to be highly significant. Without the assistance of multi-scale features, the AUC is reduced by 0.8% and 0.83% with and without the VIB module, respectively. In the absence of both neck and scale features in a network, there is a notable drop in AUC.

## 6. Conclusions and Future Work

This paper introduces a P-VIB module designed to selectively extract crucial features within a CNN-based network. Distinguishing from conventional methods that restrict feature selection solely before the classification head, our proposed approach allows feature selection at any stage within a CNN network. Experimental outcomes on the ChestX-Ray14 [5] dataset demonstrate an increase in AUC compared to state-of-the-art methods. Furthermore, the module effectively prevents overfitting, enhancing its performance when training and testing datasets are from different domains. An analysis of the feature selection loss underscores the robustness of the proposed method across various initializations, thereby enhancing its applicability across diverse domains. Last but not least, our comprehensive experiments at different dataset sizes and diverse DNN architectures point out that the P-VIB is robust and workable in many scenarios.

While the proposed method effectively mitigates overfitting on the training dataset, it falls short of bridging the gap between distinct domains. Experimental results reveal a significant decrease in the AUC metric when the AI model is applied to datasets from new domains. To advance toward a diagnostic system viable for commercial deployment, promptly addressing the domain gap between source and target domains is imperative. This can be achieved by leveraging unlabeled data and a few labeled data from the target domain for semi-supervised domain adaptation. Our future research endeavors will concentrate on developing methodologies for facilitating this adaptation process to tailor the model to a target domain rapidly.

## Figures and Tables

**Table 1:** Abbreviations of pathologies.

Pathologies	Abbreviation
Emphysema	Emph
Hernia	Hern
Nodule	Nodu
Effusion	Effu
Pleural_Thickening	PT
Pneumonia	Pne1
Cardiomegaly	Card
Infiltration	Infi
Consolidation	Cons
Atelectasis	Atel
Pneumothorax	Pne2
Mass	Mass
Edema	Edem
Fibrosis	Fibr

**Table 2:** Hyperparameter settings.

Hyperparameter	Value
Learning rate	0.0001
Patch size	64
Learning rate decay	0.9 for 2 epochs
Pre-trained on	ImageNet dataset
Optimizer	Adam
Number of epochs	20
α in Equation (1)	0.4
$\lambda_{l}$ in Equation (1)	(0.5, 0.3, 0.1) for *l* = (1, 2, 3)
Data augmentation	Resize image (256, 256); crop to (224, 224); horizontally flip (the flip probability
	is 0.5); contrast, saturation, and hue (0.9–1.1); to tensor and Normalization

**Table 3:** Methodology of the proposed method and previous works.

Method	Intrinsic Correlations	Backbone	Head	Loss	Scale
SSGE	CM	DenseNet169	FC	W-CE	S
F-PCAM	CM	DenseNet121	FC	BCE	S
CheXGAT	CM	Eff-B4	FC	FL	S
CheXGCN	CM	DenseNet169	FC	W-CE	S
MXT	ATT	ViT	FC	W-CE	S
LLAGNet	ATT	DenseNet169	MB	W-CE	S
$A^{3}$Net	ATT	DenseNet121	CNN + FC	W-CE	M
PCAN	ATT	DenseNet121	MB	BCE	S
ConsultNet	ATT	DenseNet121	FC	KL + BCE	S
PCSANet	ATT	ResNet-50	FC	BCE	M
Ours	ATT	Res2Net50	CSRA	KL + BFL	M

**Table 4:** Comparison of AUC to SotA for the ChestX-Ray14 [5] dataset. **Bold** represents the best AUC. *Italics* represent the second-best AUC.

Method	SSGE [26]	LLAG- Net [14]	CheX GCN [27]	$A^{3}$Net [13]	Cons- ultNet [15]	CheXGAT [28]	MXT [34]	PCAN [30]	PCSA- Net [31]	F-PCAM [29]	Ours
Atel	0.792	0.783	0.786	0.779	0.785	0.787	0.798	0.785	**0.807**	0.785	*0.803*
Card	0.892	0.885	0.893	0.895	0.899	0.879	0.896	0.897	**0.910**	0.897	*0.906*
Effu	0.840	0.834	0.832	0.836	0.835	0.837	0.842	0.837	**0.879**	0.837	*0.851*
Infi	0.714	0.703	0.699	0.710	0.699	0.699	*0.719*	0.706	0.698	0.706	**0.735**
Mass	0.848	0.841	0.840	0.834	0.838	0.839	*0.856*	0.834	0.824	0.833	**0.858**
Nodu	**0.812**	0.790	0.800	0.777	0.775	0.793	*0.809*	0.786	0.750	0.796	0.790
Pne1	0.733	0.729	0.739	0.737	0.738	0.741	**0.758**	0.730	0.750	0.732	*0.746*
Pne2	*0.885*	0.877	0.876	0.878	0.871	0.879	0.879	0.871	0.850	0.876	**0.887**
Cons	0.753	0.754	0.751	0.759	0.763	0.755	0.759	0.763	**0.802**	0.745	*0.776*
Edem	0.848	0.851	0.850	0.855	0.85	0.851	0.849	0.854	**0.888**	0.847	*0.874*
Emph	**0.948**	0.939	*0.944*	0.933	0.924	0.945	0.906	0.921	0.890	0.933	0.940
Fibr	0.827	0.832	0.834	0.838	0.831	*0.842*	**0.847**	0.817	0.812	0.824	*0.842*
PT	**0.795**	0.798	**0.795**	0.791	0.776	0.794	0.800	0.791	0.768	0.793	0.789
Hern	0.932	0.916	0.929	*0.938*	0.922	0.931	0.913	**0.943**	0.915	0.905	0.915
**Mean**	*0.830*	0.824	0.826	0.826	0.822	0.827	*0.830*	0.824	0.825	0.821	**0.837**

**Table 5:** Comparison of computational consumption by a single image during the test phase on the ChestX-Ray14 dataset.

Method	SSGE [26]	CheXGCN [27]	LLAGNet [14]	PCAN [30]	PCSANet [31]	Proposed Method
FLOPs (G)	17.74	17.86	34.96	2.86	3.92	7.98
Time (s)	0.059	0.061	0.094	0.054	0.072	0.049

**Table 6:** A comparison between MRFNet/P-VIB in terms of generalization for five observations. The result given by P-VIB is in *italic* format.

Training Set	Training AUC	Evaluation AUC	Testing AUC
25%	84.9/*88.36*	81.71/*82.49*	74.82/*76.81*
50%	86.96/*87.78*	83.66/*83.68*	75.87/*77.51*
70%	88.07/*88.01*	84.18/*84.20*	77.46/*79.09*
100%	89.28/*88.16*	84.13/*84.28*	78.08/*79.54*

**Table 7:** Performance with different backbones.

Dataset	100% Training Data		50% Training Data	
	**No P-VIB**	**With P-VIB**	**No P-VIB**	**With P-VIB**
Rest2Net	86.00	86.4	85.15	85.51
ResNet	84.04	85.42	82.6	83.95
DenseNet121	75.21	76.52	73.2	74.4

**Table 8:** Comparison of datasets with and without P-VIB in an ablation study on network architectures.

Dataset	100% Training Data		50% Training Data	
	**No P-VIB**	**With P-VIB**	**No P-VIB**	**With P-VIB**
No neck (N)	85.59	86.20	85.04	85.18
No multi-scale (MS)	85.17	85.60	84.94	85.05
Neither (N + MS)	84.91	85.25	84.04	84.15

## Data Availability

The data presented in this study are openly available in [https://nihcc.app.box.com/v/ChestXray-NIHCC (accessed on 20 December 2023)] at [10.1109/CVPR.2017.369], reference number [5,6].

## Abbreviations

The following abbreviations are used in this manuscript:

AUC	area under the curve
SoTA	state of the art
VIB	variational information bottleneck
DNN	deep neural network
CXR	chest X-ray
GNN	graph neural network
CNN	convolution neural network
